# Does our sleep debt affect patients’ safety?

**DOI:** 10.4103/0019-5049.76572

**Published:** 2011

**Authors:** Anurag Tewari, Jose Soliz, Federico Billota, Shuchita Garg, Harsimran Singh

**Affiliations:** Department of Anesthesiology, Dayanand Medical College & Hospital, Ludhiana, India; 1 Department of Anesthesiology & Perioperative Medicine, MD Anderson Cancer Center, Houston, TX, USA; 2 Department of Neuroanesthesiology, University of Rome, Rome, Italy; 3 Department of Critical Care Medicine, Dayanand Medical College & Hospital, Ludhiana, India

**Keywords:** Anaesthesiologist, fatigue, night shifts, patient safety, sleep debt, sleep deprivation

## Abstract

The provision of anaesthesia requires a high level of knowledge, sound judgement, fast and accurate responses to clinical situations, and the capacity for extended periods of vigilance. With changing expectations and arising medico-legal issues, anaesthesiologists are working round the clock to provide efficient and timely health care services, but little is thought whether the “sleep provider” is having adequate sleep. Decreased performance of motor and cognitive functions in a fatigued anaesthesiologist may result in impaired judgement, late and inadequate responses to clinical changes, poor communication and inadequate record keeping, all of which affect the patient safety, showing without doubt the association of sleep debt to the adverse events and critical incidents. Perhaps it is time that these issues be promptly addressed to prevent the silent perpetuation of a problem that is pertinent to our health and our profession. We endeavour to focus on the evidence that links patient safety to fatigue and sleepiness of health care workers and specifically on anaesthesiologists. The implications of sleep debt are deep on patient safety and strategies to prevent this are the need of the hour.

## INTRODUCTION

Working for extended hours predisposes to fatigue and lack of sleep. Work schedules such as night shifts or overnight call duty causes insomnia.[1] An adult requires at least 8 hours of effective sleep to avert the effects of sleep deprivation. The lack of sleep is cumulative and must be made up both in terms of longer duration of sleep as well as an increase in time spent in deep sleep. Regulatory authorities, researchers and safety experts now acknowledge the effect of sleep deprivation and exhaustion on the perioperative outcome of the patients. Considering this, the regulatory authorities in Europe and United States of America have formulated advisory guidelines for duty hours, especially for residents in training.[2]

## PHYSIOLOGY OF SLEEP

Sleep is a state of unconsciousness in which the brain is more responsive to internal than external stimuli.[3] Normal sleep is divided into non-rapid eye movement (NREM) and rapid eye movement (REM) sleep. NREM sleep begins in the lighter stages N1 and N2, and progressively deepens to slow wave sleep as evidenced by higher voltage delta waves. N3 (slow wave sleep) is present when delta waves account for more than 20% of sleep EEG. As NREM stages progress, stronger stimuli are required to result in an awakening. Stage R (REM sleep) has tonic and phasic components. The sympathetically driven phasic component is characterised by rapid eye movements, muscle twitches, and respiratory variability. Parasympathetically driven tonic REM has no eye movements. The REM period length and density of eye movement increase throughout the sleep cycle.[4] REM sleep follows NREM sleep and occurs four-five times during a normal 8 hour sleep. “Microsleep” is defined as brief (several seconds) runs of theta or delta activities that break through the otherwise beta or alpha EEG of waking.

Two primary determinants that trigger fatigue and interact dynamically are sleep homeostasis and circadian rhythms. An individual’s level of alertness or potential for sleep is determined by a multifaceted interaction of these factors. Performance and alertness decrement occurs when either of these is unsettled. Factors other than fatigue, such as workload, environment, stress, boredom, motivation, and professionalism, also influence the ability to perform. In addition, there are large inter-individual alterations on the effects of fatigue.[5] Use of alcohol or caffeine prior to bed, sleep disorders such as sleep apnoea influence the quality or duration of sleep.

Circadian sleep rhythm is modulated by the suprachiasmatic nucleus (SCN) in hypothalamus (which functions as a pacemaker) and sets the body clock. SCN receives inputs from the retina (light) and the pineal gland (secretion of melatonin). The nadir of the rhythm is early morning. Melatonin, implicated as a modulator of light entrainment, is secreted maximally during the night by the pineal gland.[6] Natural increased sleepiness occurs twice between 0300 and 0700 and 1300 and 1600 hours, and periods of maximal alertness occur at approximately 0900 to 1100 and 2100 to 2300 hours. While trying to stay awake, alert and functioning during night shifts, the normal circadian pattern is unsettled. This promotes sleep deprivation, increased fatigue, and vulnerability to judgemental errors. During the day, these individuals are unable to sleep due to the SCN stimulation by bright light. The person stays fatigued, yet awake, leading to sleep debt.

## HEALTH EFFECTS OF SLEEP LOSS

Glucose-positron emission tomography (PET) studies have shown that after 24 hours of sustained wakefulness, the metabolic activity of brain decreases significantly. Sleep deprivation results in decreased core body temperature and decrease in immune system function (measured by white cell count and activity and decreased release of growth hormone). Sleep deprivation also causes heart rate variability.[7] The areas most important for judgement, impulse control, attention, and visual association are disproportionately “hypo-metabolic” compared to the primary sensory and motor areas necessary for receiving and acting upon the environmental inputs. This finding leads to the hypothesis that the areas of brain most responsible for higher-order cognition are to some degree less functional during sleep-deprived waking activity.[8]

Microsleep is a consequence of incurring sleep debt and can occur any time. Studies which used polysomnography simultaneously proved that microsleep occurs prior to the performance failure and impaired continuity of cognitive function and may further impair performance. Often, the individual experiencing microsleep is ignorant of the transitory blackout. Other explanations for performance impairments include sensory perceptual impairment such as the development of the visual neglect phenomenon.

With decreased sleep, higher-order cognitive tasks are affected early and disproportionately. Tests requiring both speed and accuracy demonstrate considerably slow speed before accuracy begins to fail. Total sleep duration of 7 hours/night over 1 week has resulted in a decreased speed in tasks of both simple reaction time and more demanding computer-generated mathematical problem solving. Total sleep duration of 5 hours/night over 1 week shows both decrease in speed and the beginning of accuracy failure. In driving simulation tests, accidents increase progressively as total sleep duration is decreased to 7, 5 and 3 hours/night over 1 week. In the same simulations, 3 hours total sleep duration was associated with loss of ability to simultaneously appreciate peripheral and centrally presented visual stimuli, which could be termed as visual simultanagnosia and peripheral visual neglect.[9]

## THE ADVERSE EFFECTS OF SLEEP DEPRIVATION ON PERFORMANCE

In 1965, Randy Gardner intentionally stayed awake for 264 hours, the longest without sleep, for a high school project (a Guinness World Record then).[3] He experienced significant deficits in concentration, motivation, perception, and higher mental processes during his sleep deprivation. However, he recovered normal cognitive functions after a few nights of sleep. The Guinness Book of World Records has withdrawn its backing of sleep deprivation category because of the associated health risks.

A meta-analysis of 29 studies evaluated the association of physically demanding work, prolonged standing, long work hours, and cumulative “fatigue score” with preterm delivery, pregnancy-induced hypertension, and small-for-gestational-age infants. Circadian disruption alters glucose tolerance, cortisol concentrations, and sympathetic tone and affects pregnancy outcome. There was a positive association between physically demanding work and pregnancy-induced hypertension, and delivery of small-for-gestational-age infants. Shift work alone was found to increase the incidence of preterm births.[10] An increased risk of breast cancer in females who worked night shifts [odds ratio (OR) = 1.6 and relative risk (RR) = 1.36] was demonstrated in female workers[11,12] and a cohort study on female nurses.[13]

Dawson *et al*. compared subjects’ psychomotor functions during sleep deprivation and after consumption of alcohol.[13] After 17 hours of sustained wakefulness, the subject’s psychomotor performance decreased to a level that was equivalent to that when their blood alcohol concentration was 0.05%. By 24 hours of sustained wakefulness, performance decreased to the same extent as observed at a blood alcohol concentration of 0.10%, an alcohol level at which the driver of a motor vehicle is considered intoxicated in most states in the USA.[14]

Federal Transportation Agency in USA submits that sleep deprivation is a major factor in many motor vehicle accidents.[15–17] A survey conducted on emergency medicine residents indicated an increased risk of traffic accidents while driving home after a night shift.[12] A prospective, Web based survey documented the incidence of motor vehicle crashes and near-miss incidents for 2737 interns.[18] After an extended work shift (greater than 24 hours), the OR for a motor vehicle crash was 2.3 compared for travel home after a shorter shift, and was 5.9 for a near-miss occurrence. When there were five or more extended work shifts in a month, the risks that the intern would fall asleep while driving (OR = 2.4) or while stopped in traffic (OR = 3.7) were increased. In developed countries, a training programme may incur liability if one of their residents is injured in a traffic accident after working an extended shift.[19]

Studies and meta-analyses have demonstrated that after a period of sleep deprivation such as being on-call, physicians have worse language and numeric skills, poor retention of information, and impaired short-term memory and concentration.[20,21] They also observed that sleepiness has a significant negative effect on mood, with individuals demonstrating increased anger, lack of concentration, and anxiety. There are several examples of self-reported data linking the fatigue of health care personnel to medical errors. A survey of anaesthesia providers in New Zealand suggests that fatigue may have compromised patient safety with 86% of respondents reporting fatigue-related errors.[22] Because these are self-reported data, the validity of this information and the causality of fatigue in the reported errors cannot be determined. Unfortunately, some of the older published studies suffer from design flaws such as lack of control groups, inadequate documentation of sleep state, failure to control for circadian effects, and lack of randomisation.

## RESEARCH ON SLEEP DEPRIVED RESIDENTS

The State of New York implemented duty hour limits for residency training programmes, several years before national accrediting organisations addressed the issue in the USA. After regulations for resident duty hours were instituted in New York, there were no changes in in-hospital patient mortality rates, patient length of stay, or transfer rates to critical care units, but there was an increase in the rate of patient complications.[23] Another study from New York demonstrated that complications occurred more frequently when the primary resident was off duty and care was provided by cross-covering residents.[24] This suggests that the care delivered by a tired resident who knows the patient may be better than that provided a rested resident who is cross-covering and is not as familiar with a patient’s history and disease state.

Howard *et al*. assessed 11 anaesthesiology residents to determine the extent of daytime sleepiness.[25] They were tested in their baseline state (BL: while working on a general operating room rotation with no on-call period in the preceding 48 hours), in the post-call condition (PC: on the morning after a 24-hour in-hospital duty shift), and in a sleep-extended condition (EXT: in which they were instructed to maximise their sleep at night for four consecutive days and were allowed to begin work at 1000 hours each morning). The multiple sleep latency test (MSLT) was used to assess daytime sleepiness (MSLT > 10 min is normal). Sleep latency is measured as the time from lights out until there is EEG evidence of sleep. MSLT < 5 min is observed in patients with narcolepsy, obstructive sleep apnoea, and healthy individuals who have been awake for 24 consecutive hours. The subjects were also rated using the Stanford Sleepiness Scale (SSS) to determine subjective sleepiness. The SSS scale ranges from 1 to 7 with a value of 1 indicating a wide awake individual and 7 indicating that the individual feels like they are about to go to sleep. In these residents, average MSLT scores were significantly reduced in the BL (6.7 min) and PC (4.9 min) compared with EXT (12.0 min). In the PC state, test subjects were subjectively sleepier than in the other two conditions. Chronic sleep deprivation was present in the residents working their routine schedule even when tested on a day not immediately after overnight call.

A second study of anaesthesiology residents used a patient simulator in an operating room to assess the effects of sleep deprivation on psychomotor and clinical performance.[26] Twelve residents were tested in a sleep-extended (EXT) and a sleep-deprived (DEP) state (subjects were awake for at least 25 hours). The residents were videotaped while performing a 4-hour simulated anaesthetic for a laparoscopic procedure and analysed to assess the degree of sleepiness and strategies used by residents to stay awake. Residents in the DEP condition demonstrated progressive impairment of alertness, mood, and performance and had longer response latency to vigilance probes. This realistic simulation of a clinically relevant scenario demonstrated that psychomotor performance and mood were impaired and residents were visibly sleepier when in the DEP condition.

Two prospective studies of medical residents working in an ICU demonstrated adverse effects of sleep deprivation leading to serious medical errors and attention failures.[27,28] Residents working a traditional “every third night call” schedule were compared to those working a special schedule that eliminated extended work shifts (24 hours more) and reduced the total number of hours worked per week to less than 80 hours. The traditional schedule was associated with 36% more serious medical errors, 21% more serious medication errors and 5.6 times as many serious diagnostic errors compared to the special schedule.[26] Residents working the special schedule had less than half the rate of attention failures, as measured by continuous electrooculography, compared to the weeks when they were working a traditional schedule.[28]

US Department of Health and Human Services’ Agency for Healthcare Research and Quality (AHRQ) included a chapter on “Fatigue, Sleepiness and Medical Errors” within a report that assessed strategies for reducing errors and improving patient safety.[29]

## ASSESSMENT OF SLEEPINESS

Assessment of acute and chronic sleep deprivation is of paramount importance in quantifying the gravity of the problem, adding objectivity to the issue. The Epworth Sleepiness Scale (ESS) is a questionnaire intended to measure daytime sleepiness. This can be helpful in diagnosing sleep disorders.[30]

The Pittsburgh Sleep Quality Index (PSQI) was developed to measure sleep quality during the previous month and to discriminate between good and poor sleepers.[31] The PSQI is composed of 19 self-rated questions and 5 questions rated by a bed partner or roommate. The self-administered scale contains 15 multiple choice items that inquire about frequency of sleep disturbances and subjective sleep quality and 4 write-in items that inquire about typical bedtime, wake-up time, sleep latency, and sleep duration.

## STRATEGIES TO PREVENT THE ADVERSE EFFECTS OF SLEEP DEPRIVATION

In the United States, the Accreditation Council on Graduate Medical Education (ACGME) revised their policy (www.acgme.org) on duty hour requirements for all training programmes. ACGME requires training programmes to monitor duty hours of residents and must have written policies regarding duty hours and call. In-hospital work hours are now limited to 80 hours/week averaged over a 4-week cycle. It has been advised that residents must be made free from all educational and clinical duties for 24 hours/week. Training schedules must ensure that residents have adequate time for rest and personal activities. A 10-hour period must be provided between daily duty periods and after in-house call. On average, in-house call must not exceed every third night and must not exceed 24 hours. After 24 hours of in-house call, the resident may stay in the hospital on duty for six additional hours, but only for educational sessions, to transfer care of patients to other physicians, or to participate in outpatient clinics. Anaesthesia residents cannot start a new anaesthetic case in the morning after a 24-hour in-house call. When an individual has been awake for 24 hours, as might occur when on overnight call, sleep deprivation can lead to impaired psychomotor performance.

Presently, there are no duty hour limitations for anaesthesiology residents and practitioners in India and other developing countries. The same stands true for private freelancing anaesthesia practitioners who, in view of shortage of manpower and other related factors, are found working for more than 24 hours at a stretch.

It is pertinent that anaesthesiologists be involved in educational programmes dealing with the risks of sleep debt and strategies to minimise its effects. Various stratagems have been proposed to attenuate the stress of working for long sleepless hours and its detrimental effects.[32] Personnel who are expected to go for a night shift or long duty hours should be advised methods of good sleep habits prior to start of the duty. Taking a nap of at least 45 minutes is good enough to prevent sleep inertia. Other propounded methods are use of light therapy and use of caffeine during or before on-call work hours. Caffeine, widely used amongst medical professionals, must be managed as it can impact our ability to gain adequate sleep when the opportunity arises. Regular exercise can play a role, but rigorous exercise should be done at least 3 hours before sleep as it may inhibit the sleep initiation process.[33]

Modafinil, a non-amphetamine wakefulness promoting agent (originally used for excessive sleepiness from narcolepsy or obstructive sleep apnoea syndrome), has been studied by the military and may prove useful for health care workers[34] and has been recently approved for shift work sleep disorders.

The Association of Anaesthetists of Great Britain and Ireland have recommended[35] that schedules be constructed to avoid predictable fatigue and promote strategies for good sleep habits in anaesthesiologists. They encourage naps prior to extended periods of duty and recommend that hospitals have facilities for on-call personnel to sleep. They also endorse on-call responsibilities for anaesthesiologists over 55 years of age are reviewed in light of the effect of sleep deprivation.

## CONCLUSION

With a growing body of substantiation demonstrating potential untoward effects on patient safety, the issue of sleep deprivation amongst anaesthesiologists becomes vital, no more should this be ignored. Anaesthesiologists must take the requisite steps to ensure that they are meeting their own sleep demands to prevent the adverse effects of sleep debt.

Contemporary evidence on the effects of sleep restriction on neurobehavioural and physiological functioning suggests that adequate sleep duration is quintessential. No doubts should delay the implementation of urgent law formulation to prevent foreseen but unwanted mishaps. Sleep debt perpetuates neurobehavioural deficits, detrimental physiological state, obesity, cardiovascular morbidity, traffic accidents and death. It is appropriate that we work to avoid all this before it gets too late. Sleep debt impairs our ability to make decisions, handle stress, and to control our emotions. We should awaken and toil for better and judicious patient care. Let us not sleep over this anymore. Regulation and guidelines need to be formulated at national and institutional level.

It is time to wake up from our animated slumber, least we compromise our motto:“ *eternal vigilance*”

## References

[1] Åkerstedt T, Wright KP (2009). Sleep loss and fatigue in shift work and shift work disorder. Sleep Med Clin.

[2] Pickersgill T (2001). The European working time directive for doctors in training. BMJ.

[3] Stevens SM Normal sleep, sleep physiology and sleep deprivation.

[4] Iber C, Ancoli-Israel S, Chesson AL, Quan SF (2007). The AASM Manual for the Scoring of Sleep and Associated Events: Rules, Terminology, and Technical Specifications.

[5] Edgar DM, Dement WC, Fuller CA (1993). Effect of SCN lesions on sleep insquirrel monkeys: Evidence for opponent processes in sleep-wake regulation. J Neurosci.

[6] Tosini G, Pozdeyev N, Sakamoto K, Iuvone PM (2008). The circadian clock system in the mammalian retina. Bioessays.

[7] Banks S, Dinges DF (2007). Behavioral and physiological consequences of sleep restriction. J Clin Sleep Med.

[8] Desseilles M, Dang-Vu T, Schabus M, Sterpenich V, Maquet P, Schwartz S (2008). Neuroimaging insights into the pathophysiology of sleep disorders. Sleep.

[9] Thorne D, Thomas M, Russo M, Sing H, Balkin T, Wesensten N (1999). Performance on a driving-simulator divided attention task during one week of restricted nightly sleep. Sleep.

[10] Mozurkewich EL, Luke B, Avni M, Wolf FM (2000). Working conditions andadverse pregnancy outcome: A meta-analysis. Obstet Gynecol.

[11] Davis S, Mirick DK, Stevens RG (2001). Night shift work, light at night, and risk of breast cancer. J Natl Cancer Inst.

[12] Schernhammer F, Laden FE, Speizer WC, Willett WC, Hunter DJ, Kawachi I (2001). Rotating night shifts and risk of breast cancer in women participating in the nurses’ health study. J Natl Cancer Inst.

[13] Dawson D, Reid K (1997). Fatigue, alcohol and performance impairment. Nature.

[14] Lyznicki J, Doege T, Davis R, Williams MA (1998). Sleepiness, driving, and motor vehicle crashes. JAMA.

[15] White G, Spellicy M, Portmann A (2002). The chronicle of American driver and traffic safety education association.

[16] Pack AI, Pack AM, Rodgman E, Cucchiara A, Dinges DF, Schwab CW (1995). Characteristics of crashes attributed to the driver having fallen asleep. Accid Anal Prev.

[17] Steele MT, Ma OJ, Watson WA, Thomas HA, Muelleman RL (1999). The occupational risk of motor vehicle collisions for emergency medicine residents. Acad Emerg Med.

[18] Barger LK, Cade BE, Ayas NT, Cronin JW, Rosner B, Speizer FE (2005). Extended work shifts and the risk of motor vehicle crashes among interns. N Engl J Med.

[19] Pilcher J, Huffcut A (1996). Effects of sleep deprivation on performance: A meta-analysis. Sleep.

[20] Smith-Coggins R, Rosekind M, Hurd S, Buccino KR (1994). Relationship of day versus night sleep to physician performance and mood. Ann Emerg Med.

[21] Gander PH, Merry A, Millar MM, Weller J (2000). Hours of work and fatigue-related error: A survey of New Zealand anaesthetists. Anaesth Intensive Care.

[22] Laine C, Goldman L, Soukup J, Hayes JG (1993). The impact of a regulation restricting medical house staff working hours on the quality of patient care. JAMA.

[23] Petersen LA, Brennan T, O’Neil A, Cook EF, Lee TH (1994). Does housestaff discontinuity of care increase the risk for preventable adverse events?. Ann Intern Med.

[24] Howard SK, Gaba DM, Rosekind MR, Zarcone VP (2002). The risks and implications of excessive daytime sleepiness in resident physicians. Acad Med.

[25] Howard SK, Gaba DM, Smith BE, Weinger MB, Herndon C, Keshavacharya M (2003). Simulation study of rested versus sleep-deprived anesthesiologists. Anesthesiology.

[26] Landrigan CP, Rothschild JM, Cronin JW, Kaushal R, Burdick E, Katz JT (2004). Effect of reducing interns’ work hours on serious medical errors in intensive care units. N Engl J Med.

[27] Lockley SW, Cronin JW, Evans EE, Cade BE, Lee CJ, Landrigan CP (2004). Effect of reducing interns’ weekly work hours on sleep and attentional failures. N Engl J Med.

[28] Howard SK, Rosekind MR, Katz JD, Berry AJ (2002). Fatigue in anesthesia: Implications and strategies for patient and provider safety. Anesthesiology.

[29] Wachter RM, McDonald KM Making Health Care Safer: A Critical Analysis of Patient Safety Practices. Evidence Report/ Technology Assessment, No. 43. AHRQ Publication No. 01- E058, July 2001. Agency for Healthcare Research and Quality.

[30] Johns MW (1991). A new method for measuring daytime sleepiness: The Epworth sleepiness scale. Sleep.

[31] Buysse DJ, Reynolds CF 3rd, Monk TH, Berman SR, Kupfer DJ (1989). Psychiatry Res.

[32] (2001). Committee on Military Nutrition Research Food and Nutrition Board. Caffeine for the sustainment of mental task performance. Institute of Medicine.

[33] Howard SK (2005). Sleep deprivation and physician performance: Why should I care?. Proc (Bayl Univ Med Cent).

[34] Sherman BW, Strohl KP (2004). Management of shift worker sleep disorder: Alice in Wonderland redux?. J Occup Environ Med.

[35] Jha AK, Duncan BW, Bates DW (2001). Fatigue, sleepiness and medical errors. Making Health Care Safer: A Critical Analysis of Patient Safety Practices. Agency for Healthcare Research and Quality; U.S. Department of Health and Human Services.

